# Reducing Differential Item Functioning via Process Data

**DOI:** 10.1017/psy.2025.10072

**Published:** 2025-12-10

**Authors:** Ling Chen, Susu Zhang, Jingchen Liu

**Affiliations:** 1 Statistics, Columbia University in the City of New York, USA; 2 Psychology, University of Illinois at Urbana-Champaign, USA; 3 Statistics, University of Illinois at Urbana-Champaign, USA; 4 Statistics, Columbia University, USA

**Keywords:** differential item functioning, item response theory, process data, scoring rule

## Abstract

Test fairness is a major concern in psychometric and educational research. A typical approach for ensuring test fairness is through differential item functioning (DIF) analysis. DIF arises when a test item functions differently across subgroups that are typically defined by the respondents’ demographic characteristics. Most of the existing research focuses on the statistical detection of DIF, yet less attention has been given to reducing or eliminating DIF. Simultaneously, the use of computer-based assessments has become increasingly popular. The data obtained from respondents interacting with an item are recorded in computer log files and are referred to as process data. In this article, we propose a novel method within the framework of generalized linear models that leverages process data to reduce and understand DIF. Specifically, we construct a nuisance trait surrogate with the features extracted from process data. With the constructed nuisance trait, we introduce a new scoring rule that incorporates respondents’ behaviors captured through process data on top of the target latent trait. We demonstrate the efficiency of our approach through extensive simulation experiments and an application to 13 Problem Solving in Technology-Rich Environments items from the 2012 Programme for the International Assessment of Adult Competencies assessment.

## 1 Introduction

Ensuring test fairness in educational and psychometric assessments has been a major concern for researchers. A typical approach to ensuring test fairness is through differential item functioning (DIF; Holland & Wainer, 2012) analysis. DIF occurs when an item’s response function depends on not only the target latent trait to be measured by the item, but also the respondents’ group memberships that are often linked to their demographic characteristics. When DIF is present, the measurement properties of the item differ systematically across groups, leading to measurement bias (Millsap, 2012).

The research on DIF predominantly focuses on the statistical detection of its existence. DIF detection with manifest groups is typically categorized as non-parametric (Cao et al., 2017; Dorans & Holland, 1992; Dorans & Kulick, 1986; Holland & Thayer, 1986; Woods et al., 2013; Zwick et al., 2000) and parametric (Lord, 1977, 1980; Raju, 1988; Rudner et al., 1980; Swaminathan & Rogers, 1990; Thissen et al., 2013). When the comparison groups are unavailable, latent DIF analysis compares how the item functions different across latent groups (Cho & Cohen, 2010; Cho et al., 2016b; De Boeck et al., 2011). More recently, DIF detection methods that do not require predefined group memberships or anchor items have been proposed (Chen et al., 2023; Halpin, 2024; Ouyang et al., 2025; Wallin et al., 2024). While research has explored methods on handling items with DIF (Cho et al., 2016a; Liu & Jane Rogers, 2022), items identified with significant DIF are often removed during item calibration, leading to wasted resources and efforts in their development and administration. Consequently, there has been growing interest in reducing or eliminating DIF, as well as understanding the underlying reasons for why DIF occurs (Ackerman & Ma, 2024).

One way to attribute the cause of DIF is multidimensionality, where DIF arises due to the presence of secondary dimensions in the latent space (Ackerman, 1992; Kok, 1988; Shealy & Stout, 1993). Ideally, differences in the response probabilities solely reflect variations in the latent ability that the item is designed to assess, which is the primary dimension. Secondary latent traits with heterogeneous distributions across subpopulations may also contribute to these differences. These secondary dimensions are called auxiliary if they are intentionally measured by the item, or nuisance otherwise (Roussos & Stout, 1996). In our examples, computer proficiency is one of such nuisance traits correlated with problem-solving strategies, response time, and the final responses. As we will show in the PIAAC data analysis, age is one of the background variables having substantial DIF for multiple items, part of which is due to differences in computer proficiency among age groups.

The multidimensional IRT (MIRT) model has been used to analyze DIF, where both the target trait and the nuisance trait are used to model item response probabilities (Ackerman, 1992; Ackerman & Ma, 2024; Shan & Xu, 2024; Wang, Zhu, et al., 2023). Latent DIF models have also been used to investigate the secondary dimensions, using mixture models to identify latent groups as the secondary dimension and associating it with examinees’ demographic characteristics (Cohen & Bolt, 2005; De Boeck et al., 2011). The multiple-indicator–multiple-cause (MIMIC) model provides another approach from the dimensionality perspective, although only the primary dimension is used (De Boeck et al., 2011). In a mediated MIMIC model proposed in Cheng et al. (2016), a secondary dimension construct (the scale of self-confidence) is used as a potential mediator. Despite these advances, studies that rely only on response data face challenges, particularly when prior knowledge of nuisance traits is limited. Thus, it is of interest to identify the secondary dimensions from data sources beyond the response outcome data.

One data source that presents new opportunities for identifying secondary latent dimensions in DIF analysis is process data (He et al., 2021; Li et al., 2024; Wang, Tang, et al., 2023). Process data capture the problem-solving processes as respondents interact with computer-based test items. The respondents’ actions are logged as time-stamped action sequences in computer log files, making process data a detailed record of respondents’ behaviors. Three prominent examples of process data are from the Programme for International Student Assessment (PISA; e.g., OECD, 2012c), the Programme for the International Assessment of Adult Competencies (PIAAC; e.g., OECD, 2012a), and the National Assessment of Educational Progress (NAEP; e.g., Bergner & von Davier, 2019). These assessments not only measure skills traditionally tested with paper-and-pencil methods but also evaluate more complex abilities, such as Problem Solving in Technology-Rich Environments (PSTRE). Compared to traditional outcome data that are typically dichotomous (correct/incorrect) or polytomous (partial credit), process data provide more comprehensive information about the respondents’ behaviors toward completing tasks. Process data have been proven useful for accurate assessment (Zhang et al., 2023), process-incorporated measurement models (Chen, 2020; Liang et al., 2023; Tang, 2024; Xiao et al., 2021; Xiao & Liu, 2024), strategy and behavioral pattern analysis (Gao et al., 2022; He et al., 2021, 2023; Ulitzsch et al., 2022), etc. Process data are potentially a good data source to identify nuisance traits. For example, engagement is an acknowledged nuisance trait (Wise & DeMars, 2006; Wise & Kong, 2005), and is usually measured by total response time or total number of actions (Sahin & Colvin, 2020). The first principal component of features extracted from the 2012 PIAAC process data is highly correlated with engagement (Tang, Wang, He, et al., 2020). If we are able to identify the secondary dimensions that lead to DIF, we could reduce or potentially remove DIF by appropriately incorporating such a dimension in the scoring rule.

In this article, we propose a novel method for reducing DIF that leverages process data and introduce the corresponding scoring rule that only depends on the respondents’ behaviors. We attribute DIF to multidimensionality and discuss the method within the framework of generalized linear models (GLMs). We assume there is a reasonably accurate estimate of the target latent trait (primary dimension). The nuisance trait is partially predictable by the process data. The key innovation of our approach lies in constructing a surrogate for the nuisance trait using features extracted from process data. This surrogate is formulated as a linear combination of the process data features, with the weights determined by minimizing the maximum-likelihood difference between models with and without the grouping variable. The motivation is to construct a measurement model with the target trait and nuisance trait surrogate, while minimizing the impact of the grouping variable on the measurement model. We show that in the simple case of a linear model (classical test theory), the proposed optimization problem has a closed-form solution. For generalized linear models, the optimization can be solved by well-established numerical methods. We propose a new scoring rule that incorporates both the target latent trait and nuisance trait surrogate that reduces DIF. We stress that the scoring rule is purely based on respondents’ responses. We further propose an iterative method to reduce the dependence on the initial target trait estimate. The effectiveness of this method is demonstrated through simulation studies and a case study.

The rest of the article is structured as follows. Section 2 outlines the methodology of the proposed approach, while Section 3 presents the results of the simulation experiments. In Section 4, we demonstrate a case study using the PIAAC 2012 dataset. Finally, we provide a discussion in Section 5.

## 2 Method

Consider *N* independent respondents and their process and outcome responses to one item of interest. Let 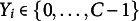 represent the response of the *i*-th respondent, where *C* is the number of possible responses. For example, when 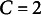 , 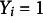 indicates correct response and 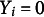 indicates otherwise. We use 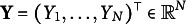 to denote the vector of responses from all *N* respondents. We introduce one grouping variable 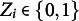 , where 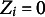 represents the reference group and 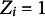 represents the focal group. The item is assumed to measure a unidimensional latent trait, denoted by 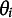 for respondent 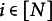 . Let 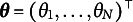 be the collection of latent traits of all respondents. Additionally, we extract process features 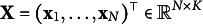 from the action sequences of the process data with the multidimensional scaling procedure proposed in Tang, Wang, He, et al. (2020), where 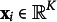 . Since process features capture most of the useful information of process data when the feature dimension is sufficiently large (Tang, Wang, He, et al., 2020; Tang, Wang, Liu, et al., 2020), we will use process features as proxies for the original process data in this work.

### 2.1 Differential item functioning

We adopt a multidimensionality-based DIF framework. In addition to the target trait dimension, 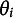 , a nuisance trait dimension, 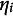 , also influences the probability of the response. Denote 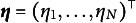 as the vector encompassing the nuisance traits for all respondents. DIF occurs when the item response probability depends on the nuisance trait, and there is a distributional difference of the nuisance trait among the two groups. Conditional on 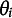 , 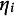 , and 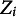 , it is assumed that 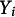 are independently distributed. When the distribution of 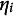 conditional on 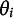 differs across the two subgroups, we have 

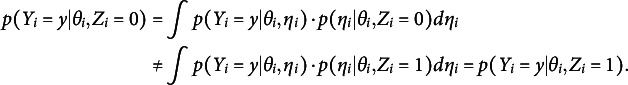

Therefore, under this multidimensional framework, a unidimensional measurement model leads to DIF.

Specifically, we consider a GLM with the following conditional distribution: 
(1)

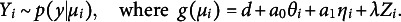

Here, 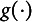 is the link function with respect to the response mean 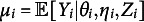 , and 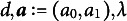 are unknown coefficients. When 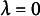 , the distributional difference of 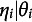 among the two subgroups is the only DIF source in a unidimensional measurement model. The model specified by (1) is quite general, as we allow 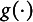 to take a general form for a wide range of response types, such as binary, polytomous, and continuous responses. For binary responses, the logistic regression model with 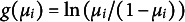 is referred to as the multidimensional two-parameter logistic (M2PL) IRT model. In addition, 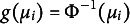 corresponds to the probit regression model with 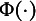 being the cumulative distribution function of the standard normal distribution. When 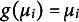 , the model becomes a linear regression model for continuous responses.

### 2.2 DIF reduction

One of the primary challenges in applying the multidimensionality-based DIF analysis is that the nuisance trait, 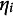 , is unobserved and is generally difficult to be directly measured. We propose to construct a surrogate for the unobserved nuisance trait and incorporate it in the item response function to correct DIF. Specifically, we aim to build a surrogate, 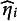 , such that DIF is only attributed to the distributional differences in 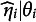 , and the conditional probabilities of item responses become approximately equal across different subgroups. Formally, we achieve the following: 
(2)

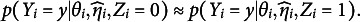



We propose using process data to construct a nuisance trait surrogate, driven by two key motivations. Firstly, process data capture the entire sequence of actions taken by each respondent as they interact with and solve an item, providing a rich source of information on various nuisance traits. Second, process data typically predict the final response with perfect accuracy. By analyzing the respondent’s full sequence of actions, we can infer whether they answered the item correctly or their partial scores. In theory, adding all available process features in the model eliminates DIF. Yet this approach is not ideal as the resulting measurement model solely depends on the process features and provides little information of the target trait.

The core of our proposed method is to identify an optimal linear combination of process features as a surrogate for the nuisance trait 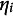 . Specifically, we consider 

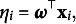

where 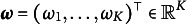 is the weight vector and we assume 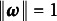 for model identifiability. Our objective in identifying 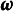 is to minimize a quantity equivalent to the likelihood ratio test statistic, 
(3)

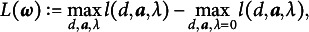

where 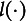 is the log-likelihood function, 
(4)

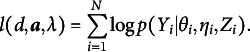



Function (3) quantifies how much model fit is increased after adding the grouping variable into the model, thus can be viewed as a quantification of the DIF effect. When there is no DIF exhibited, adding the grouping variable into the model would barely increase the likelihood, and we would expect 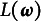 to be close to 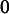 . This objective function enables us to optimize 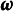 by comparing models with and without the grouping variable, ultimately reducing or removing DIF. Therefore, we propose the estimation of 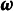 to be the minimizer of the objective function 
(5)

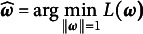

and the nuisance trait surrogate is 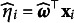 . We will show that the solution of (5) has a closed-form solution in the linear regression model with one grouping variable (see Section 2.3). In other cases, we can optimize the objective function numerically.

**Figure 1:**
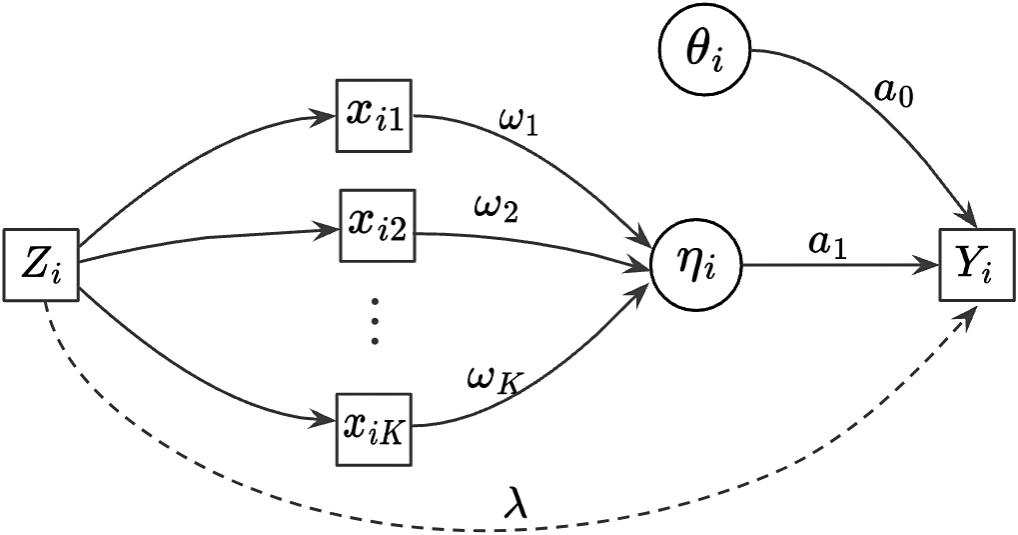
Measurement model without the intercept.

Figure 1 displays the updated measurement model incorporating both the nuisance trait and target trait. With the nuisance trait surrogate, we update the initial estimate of the target trait with the two-dimensional measurement model. Consider the case with *J* items, among which items 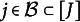 exhibit DIF. Suppose the nuisance trait surrogates 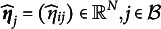 have been estimated for the DIF items, then we obtain the maximum-likelihood estimate (MLE) of the item parameters 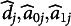 with 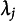 fixed as 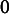 in (1) for 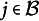 , and 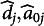 with 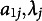 fixed as 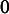 for 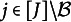 . The target trait estimate is then updated by the MLE 
(6)

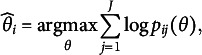

where 

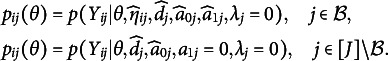

### 2.3 A special case: Linear model with closed-form solution

When the link function *g* is the identity function, model (1) becomes the linear regression model. As DIF is defined as the group difference of the distribution of *Y* conditional on the latent trait, we conduct our DIF analysis in the orthogonal subspace of 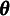 in the linear model. To be more specific, we consider the residuals of 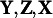 after regressing on 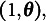 respectively, denoted by 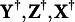 .

The model with and without the grouping variable can be rewritten as a reduced and a full linear regression model 
(7)

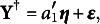



(8)

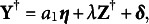

where 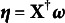 . If we assume 
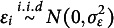
 and 
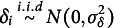
 , the objective function (3) is equivalent to 
(9)

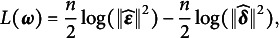

where 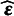 and 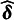 are the linear regression residuals in (7) and (8). The zero of the objective (9) turns out to have a closed-form expression under some weak conditions, in which case DIF can be fully removed. Without loss of generality, we assume that all the features are orthogonal and scaled, i.e., 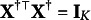 . This is achieved by principal component analysis in practice. We also assume that 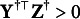 .

Proposition 1. Assume that 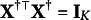 . Let 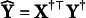 and 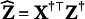 . Under the condition that 
(10)

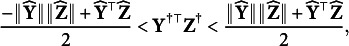

there exists 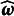 such that 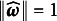 and 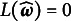 . Specifically, denote 

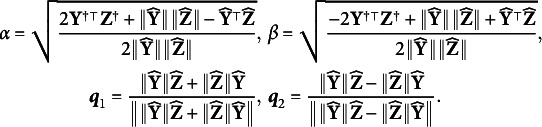

Then, 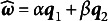 or 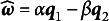 satisfies 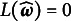 .

We note that 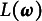 has multiple zeros, as established in Proposition 1. We choose 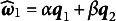 over 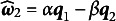 for the following reason. When 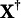 predicts 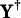 with high accuracy, 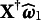 aligns with the projection of 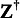 onto the column space of 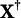 , whereas 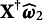 aligns with the projection of 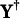 onto the same subspace. As the goal is to reduce DIF, we choose to use 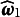 over 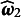 . For further details, see the proof of Proposition 1 in the Appendix.

There are several scenarios in which condition (10) holds. The first scenario occurs when 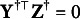 , which arises if the response and the grouping variable are independent conditional on the target trait, indicating that the item is not a DIF item, and condition (10) is automatically satisfied. The second scenario is when 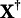 has high linear predictability of 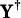 . Specifically, when 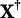 predicts 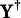 with high accuracy, we find that 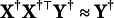 , which leads to 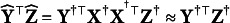 , making it straightforward to verify condition (10). This is the case where process data capture the nuisance factors that affect the response. In the simplest case, we assume 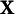 can linearly predict 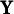 with full accuracy and let 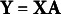 with some vector 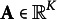 . Then, 

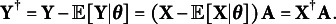

Accordingly, we consider this case to be achievable. The third scenario is when 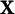 has high linear predictability of 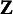 . Following similar calculations, we conclude that condition (10) is satisfied. With that being said, it is generally difficult to predict 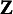 with process data.

### 2.4 General cases

The main focus of this article is to reduce the DIF of univariate *Z*. Nonetheless, we have a brief extension to some simple cases of multi-dimensional *Z*. While we have previously focused on uniform DIF in a linear factor model with one grouping variable, the proposed method is applicable to other general cases. In what follows, we will shift our focus to addressing non-uniform DIF, continuous covariates, multiple grouping variables, and nonlinear models. The goal is to minimize the objective function (3).

#### 2.4.1 Non-uniform DIF

Non-uniform DIF occurs when not only the intercept parameter, but also the discrimination parameter (the coefficient of 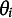 ) differs across groups. More specifically, for non-uniform DIF, (1) becomes 
(11)

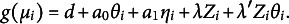

Non-uniform DIF can be viewed as a special case involving one grouping variable 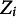 and a continuous covariate 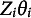 . Therefore, non-uniform DIF is included in the continuous covariates and multiple-group cases discussed below.

#### 2.4.2 Continuous covariates

In some applications, DIF is brought by continuous covariates. For instance, in computer-based tests, age is a very important variable. As our proposed method does not require 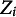 to be a discrete variable, it is applicable when 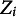 is a continuous variable. To see this, let 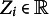 , and we aim to construct 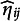 such that 
(12)

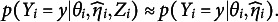

When (12) holds, we expect the objective function (3) to be close to 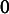 . Therefore, minimizing (3) to reduce DIF is valid for continuous covariates.

#### 2.4.3 Nonlinear models

When the link function 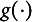 is not linear, e.g., the M2PL model and the Probit regression model, minimizing the objective function (3) is a nested optimization problem that takes in different forms depending on the model employed. Again, we rely on numerical methods to approximate the solution.

#### 2.4.4 Multiple grouping variables

Sometimes, it is of interest to evaluate DIF over more than one grouping variable (Bauer et al., 2020; Kim et al., 1995). For cases involving multiple grouping variables, the expression of the objective function in terms of 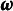 becomes significantly more complex. To address this, we propose an approximation of the objective function for *M* grouping variables 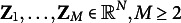 as follows: 
(13)

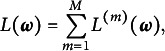

where 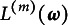 is the objective function (3) corresponding to 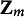 . In general, there is no closed-form solution for the minimizer of (3) or (13) with the presence of multiple grouping variables. Therefore, we rely on numerical methods to approximate the minimizer.

### 2.5 Procedure

We outline the procedure of the proposed method in a practical setting with *J* items.

1. Suppose we have access to a set of anchor items that are DIF-free, which are used to perform DIF detection on all the items. Suppose DIF has been detected on a subset of items 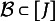 .
2. Obtain an initial latent trait estimate 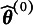 using the items without DIF.
3. For each item, perform the proposed method to obtain the nuisance trait surrogates and DIF-corrected model parameters. More specifically, for each item 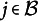 ,
  - With the initial ability estimate, 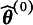 , find the minimizer 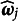 in Equation (5) and obtain the nuisance trait estimate 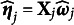 .
  - With 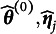 , obtain the item parameter estimates 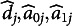 in (1) with 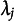 set to 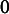 , using the full data.
4. Obtain the updated estimate of 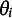 with (6), utilizing the nuisance trait surrogates and the calibrated measurement models, denoted by 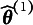 .

The above procedure requires an initial estimate 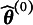 that does not have DIF. In case that DIF-free items are not available, we could apply the above method iteratively. In particular, if 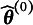 is known to have some DIF, we apply the above procedure (steps 1–4) and obtain 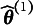 . Generally speaking, 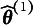 has less DIF than 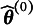 . We set the updated estimate 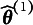 the initial value and apply steps 1–4 and obtain 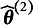 . We could iteratively apply the procedure until some type of convergence is reached. The convergence could be measured by some difference (or similarity) metric between two iterations: 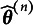 and 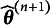 . In our real data example, we used the sample correlation and two iterations are sufficient. The correlation between 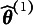 and 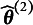 is over 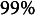 . Researchers may use other metrics depending on the specific scope of their study, such as average difference, maximum difference, general 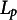 distance, etc.

## 3 Simulation studies

We carry out extensive simulation experiments to evaluate the proposed method in this section. The goal is to show that the proposed method is able to minimize the objective function, accurately estimate the nuisance traits and the item parameters, and correct for target trait estimation from the DIF items.

### 3.1 Simulation settings

We consider three settings with the sample sizes 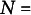 2,000, 5,000, and 10,000. Among the subjects, 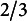 are in the reference group and 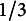 are in the focal group. We fix the number of items as 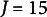 and consider low, medium, and high proportions of DIF items, that is, 3, 5, and 10 DIF items. We also consider two settings for the DIF parameters 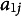 . For small DIF effects, 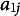 s are uniformly sampled from 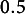 to 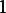 ; for large DIF effects, the range is from 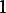 to 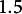 . In summary, there are 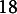 simulation settings varying in sample size, proportion of DIF items, and DIF effect size. For each simulation setting, 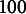 independent replications are generated. In each replication, we independently sample the target latent trait 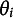 from 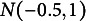 for the focal group, and 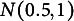 for the reference group. The difficulty parameters 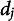 are sampled uniformly from 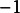 to 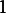 and the discrimination parameters 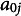 are sampled uniformly from 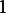 to 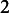 . As the generation of process data is challenging, we generate the process data features directly. The number of process data features for each item is fixed at 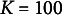 . The process data features 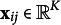 are first independently sampled from the multivariate Gaussian distribution 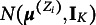 . The mean of the process features depends on the respondent’s group membership. For the reference group, 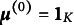 , and for the focal group 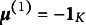 . We then right multiply 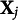 with sample 
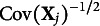
 for the process features to have the identity matrix as the covariance. The ground-truth nuisance trait is a linear combination of the process features: 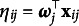 , with 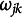 sampled independently from the exponential distribution with rate 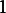 and 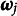 ’s are scaled to have unit norm. Note that the generated nuisance traits for each item have unit norm. We consider both the linear model and the M2PL model in generating the item responses. As DIF is solely introduced by the distributional difference of the nuisance trait in simulation, 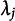 is set to be 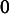 in (1) when generating the item responses for both models.

For the linear factor model, we set the variance of the item response noise as 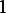 when generating the item responses. To make sure that the features can almost perfectly predict each item response in the linear model, we add one column of 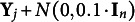 to the generated 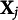 for each item *j* and generate the nuisance traits with the same manner as mentioned above. The initial target trait estimates 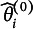 are obtained with factor analysis using responses from the DIF-free items. To construct the nuisance trait, we adopt the closed-form expression of 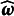 from Proposition 1. For the M2PL model, we generate 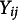 with the link function 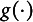 being the logit function. The initial target trait estimates 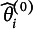 are the MLE estimates using the DIF-free items. The nuisance traits are estimated by solving (5) using the optim function with the L-BFGS-B optimizer in R.

The simulation above corresponds to uniform DIF. In addition, we consider the case with non-uniform DIF so that 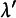 in (11) is not zero. Similar simulation settings are adopted except for the generation of process data features. To ensure the existence of non-uniform DIF, the process features 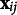 are sampled from the multivariate Gaussian distribution 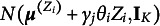 with 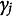 simulated from the exponential distribution with rate 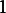 .

### 3.2 Evaluation criteria

We consider five evaluation criteria. Firstly, we check whether the proposed method is able to reduce the objective function value. Specifically, we verify whether zero of (3) is obtained for the linear model with Proposition 1. Secondly, we evaluate the correlation of nuisance trait estimation compared to its ground truth. Thirdly, we calculate the mean squared error (MSE) of the item parameter estimations. In addition, to evaluate measurement reliability after changing the scoring rule, we calculate the Fisher information (FI) of the target trait for the DIF items. For the linear model, target trait FI for the item 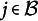 is 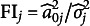 , where 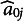 is the estimated coefficient for the target trait and 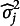 is the estimated variance. For the M2PL model, 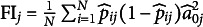 , where 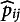 is the estimated response of the logistic model. Last but not least, we consider the between-group sum of squared (SS) bias for target trait estimation using the DIF items. We elaborate more on this evaluation criterion assuming one grouping variable with a focal group and a reference group. Note that this criterion can be easily generalized to multiple grouping variables. For any target trait estimate 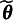 , define the bias 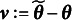 and denote the mean of bias as 
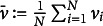
 . Consider the between-group SS for the bias of 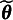 in analysis of variance (ANOVA): 
(14)

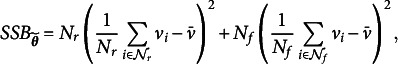

where 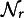 and 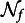 are the index sets of the reference group and focal group, respectively, and 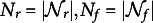 . To illustrate that the proposed method is able to de-bias target trait estimation, we compare the above-defined value for two estimates. The benchmark estimate is the MLE computed using the responses from the DIF items in 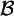 , assuming DIF is not present: 

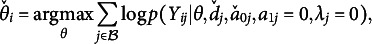

where 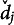 and 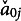 are calibrated on the DIF items. The second estimate is the MLE computed after DIF-correction: 

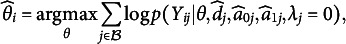

where 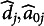 and 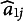 are calibrated on the DIF items with the nuisance trait surrogates. Because of the presence of DIF, 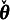 is expected to be over-estimated within one group, and under-estimated within the other, leading to large values of 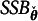 . With the proposed method, we expect 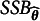 for the corrected estimate to be small compared to 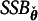 for the not-corrected estimate.

### 3.3 Simulation results

**Figure 2:**
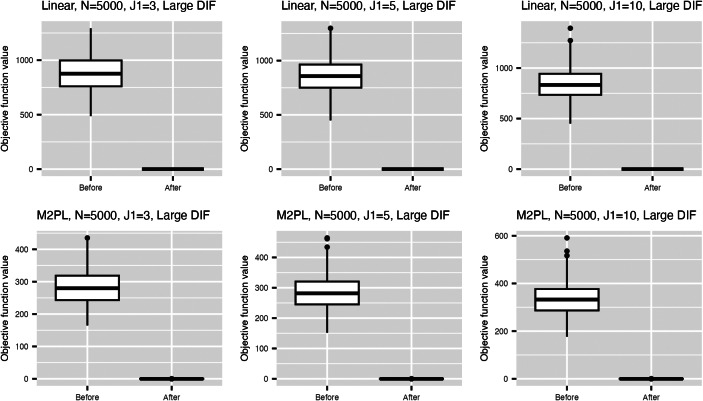
Values of the objective function before and after adding the nuisance trait surrogate for the linear model (upper) and the M2PL model (lower) with *uniform* DIF, 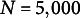 , and large DIF.

**Figure 3:**
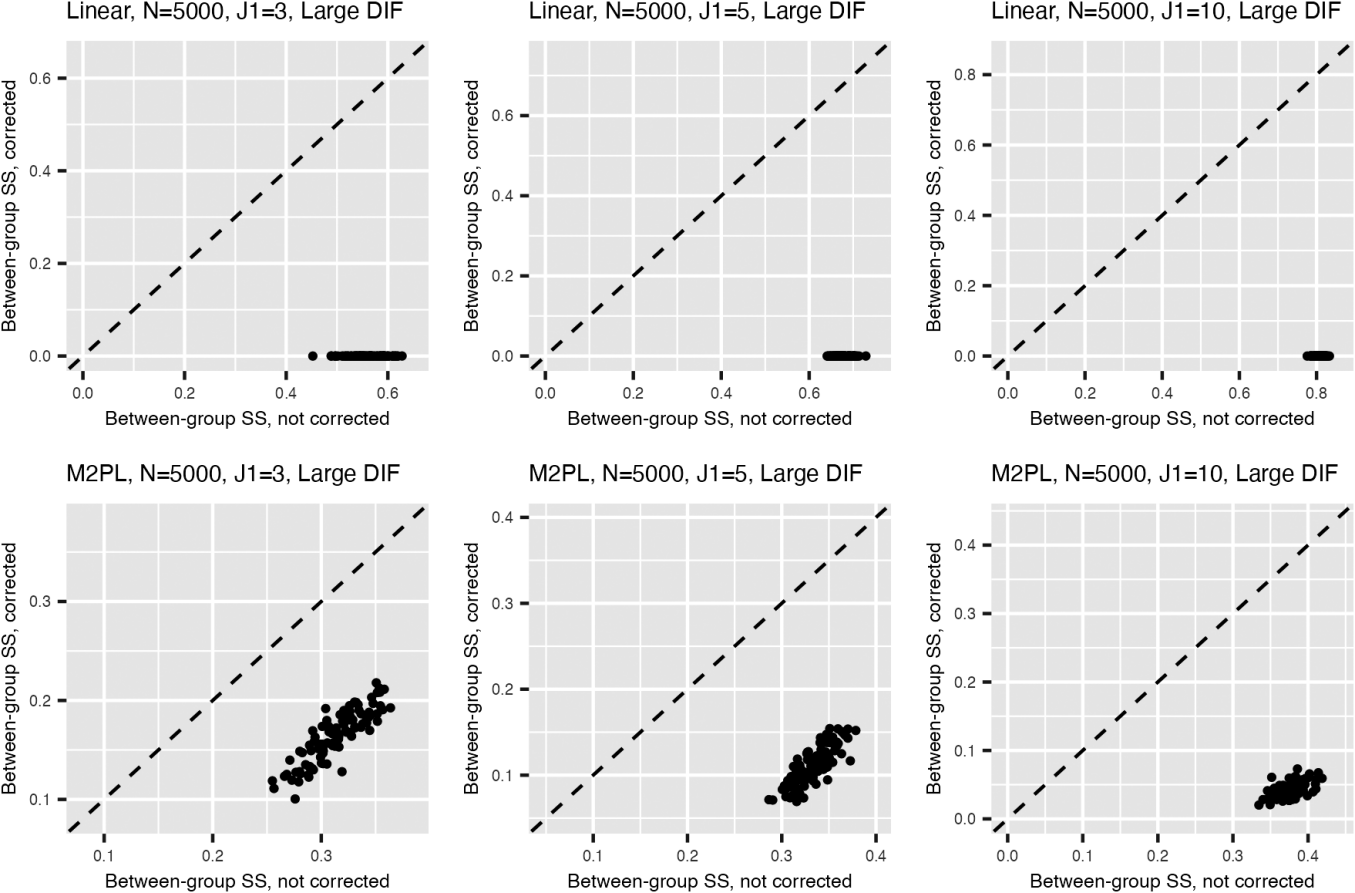
Between-group sum of squared bias for target trait estimation for the linear model (upper) and the M2PL model (lower) with *uniform* DIF, 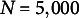 , and large DIF. The *x*-axis corresponds to the estimation without DIF correction using the DIF items; the *y*-axis corresponds to the DIF-corrected estimation using the DIF items.

**Figure B1:**
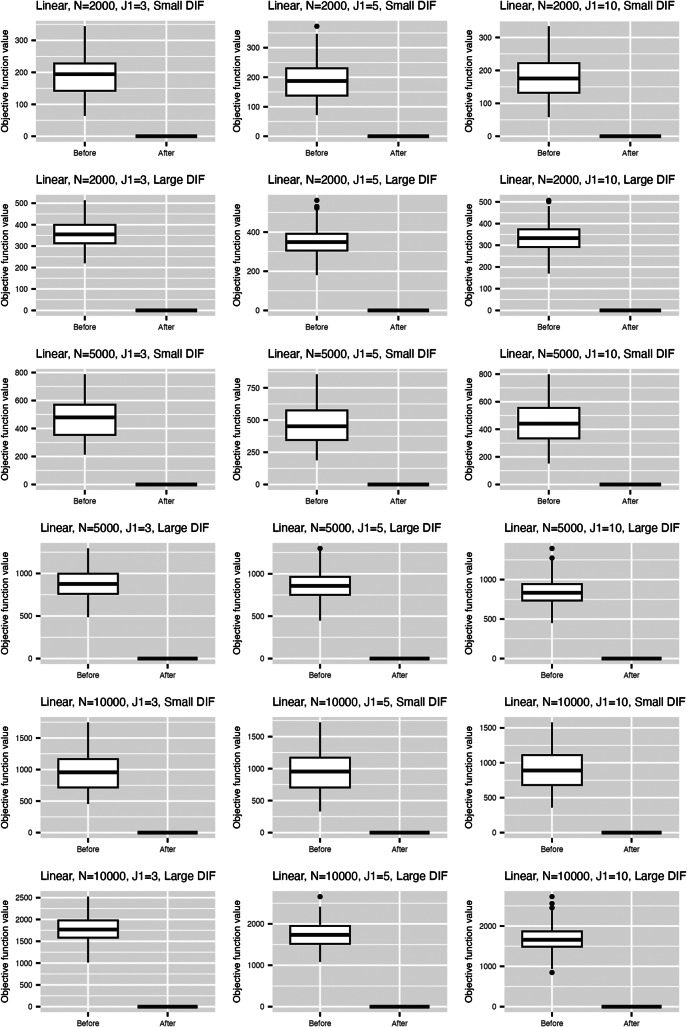
Objective function value before and after adding the nuisance trait surrogate for the *linear model* with *uniform* DIF under different simulation settings.

**Figure B2:**
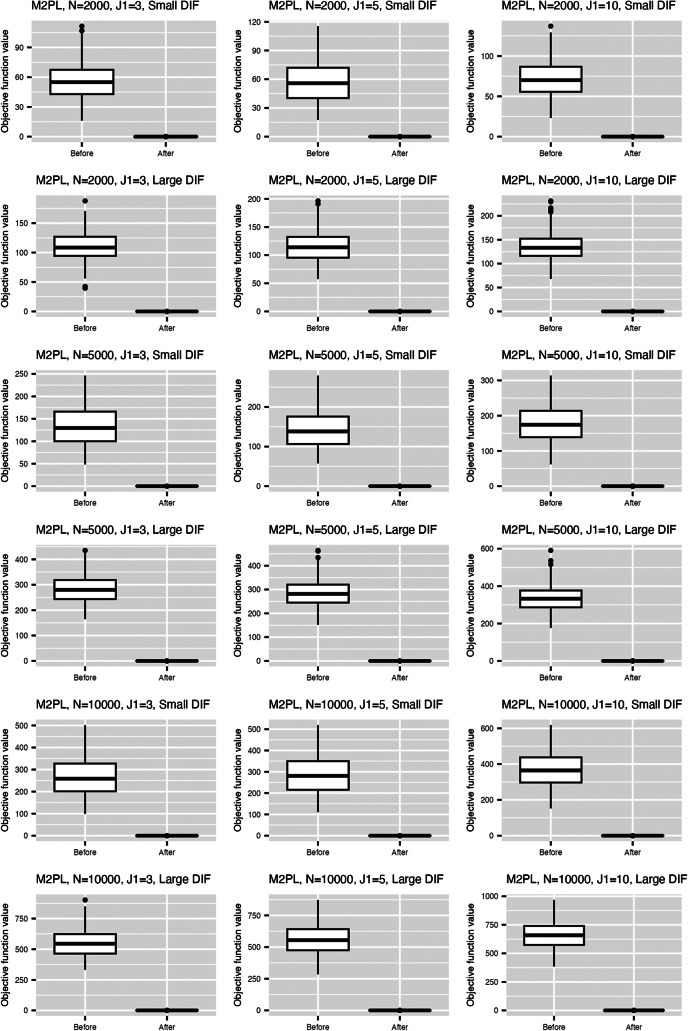
Objective function value before and after adding the nuisance trait surrogate for the *M2PL model* with *uniform* DIF under different simulation settings.

**Figure B3:**
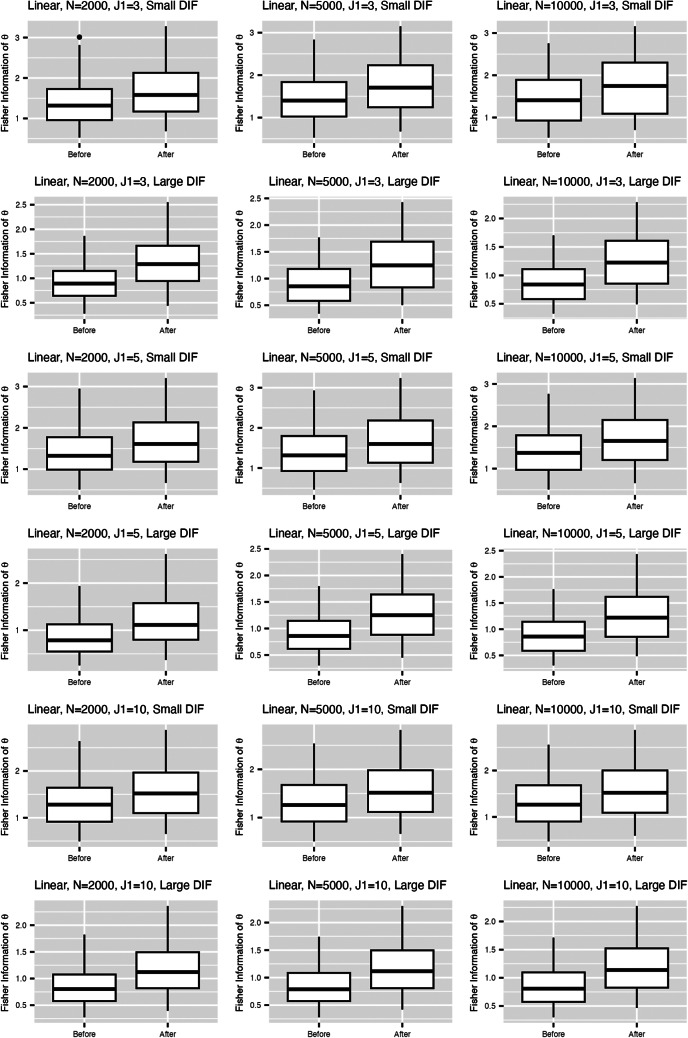
Fisher information of 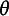 in the measurement model before and after adding the nuisance trait surrogate for the *linear* model with *uniform* DIF under different simulation settings.

**Figure B4:**
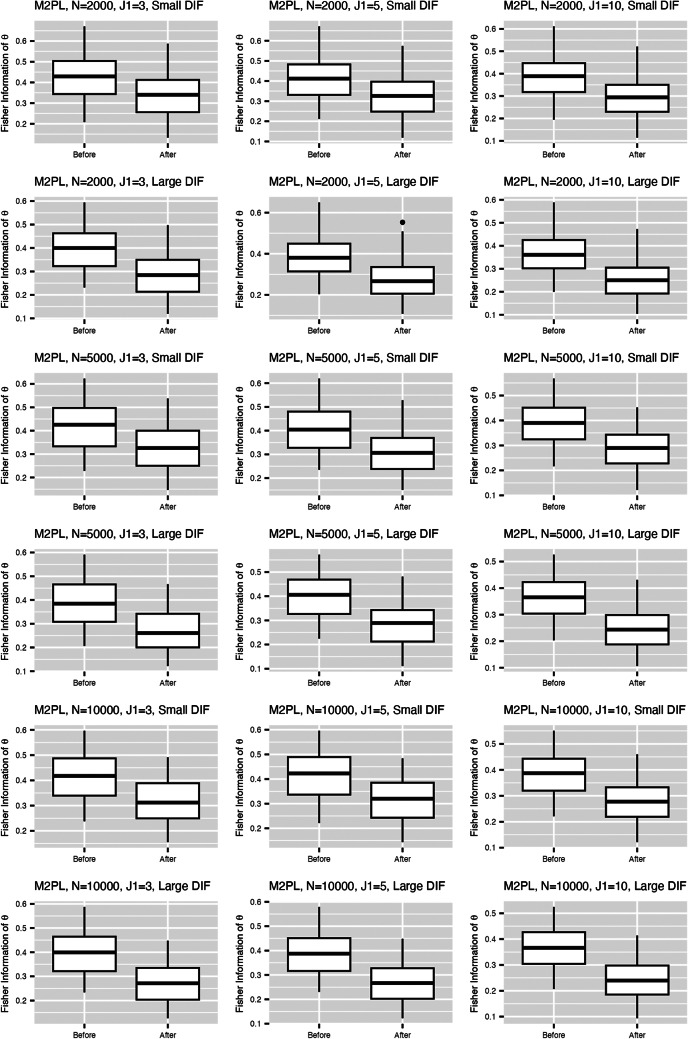
Fisher information of 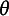 in the measurement model before and after adding the nuisance trait surrogate for the *M2PL* model with *uniform* DIF under different simulation settings.

**Figure B5:**
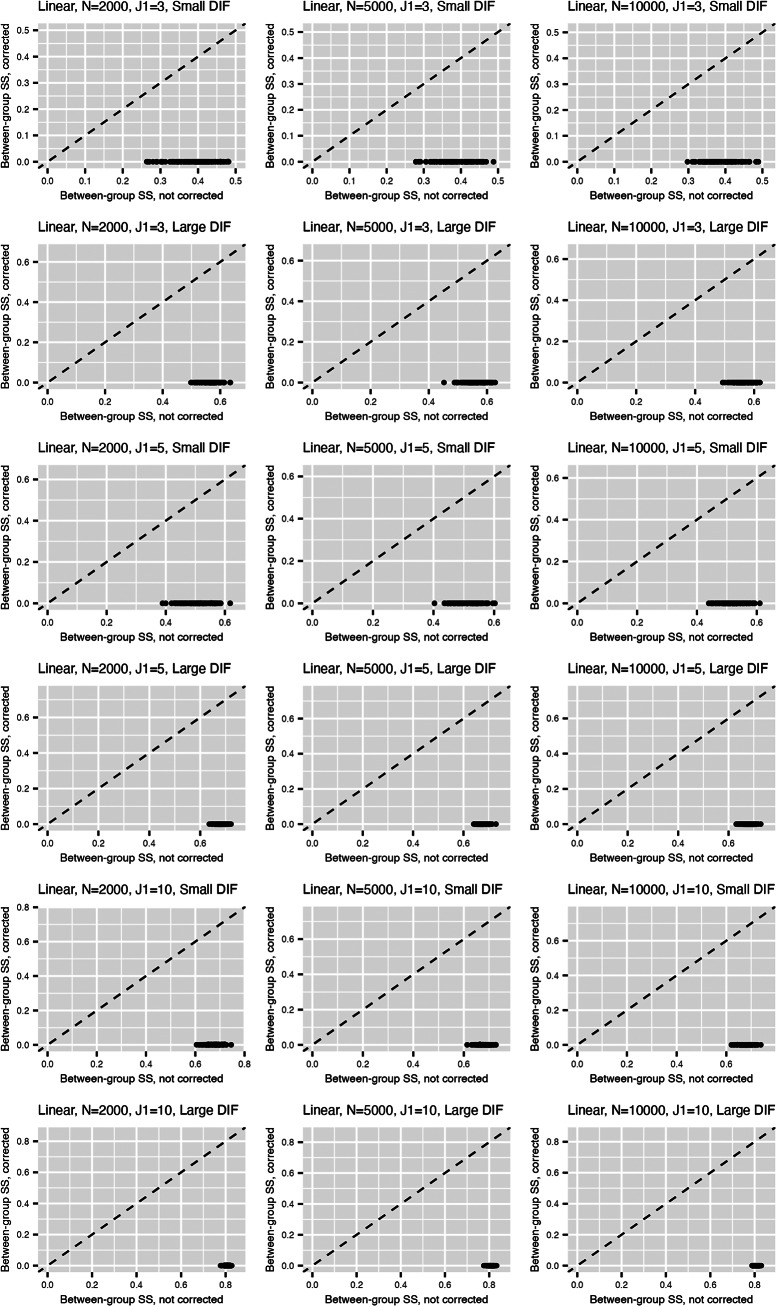
Between-group sum of squared bias for target trait estimation for the *linear* model with *uniform* DIF under different simulation settings. The *x*-axis corresponds to the estimation without DIF correction using the DIF items; the *y*-axis corresponds to the DIF-corrected estimation using the DIF items.

**Figure B6:**
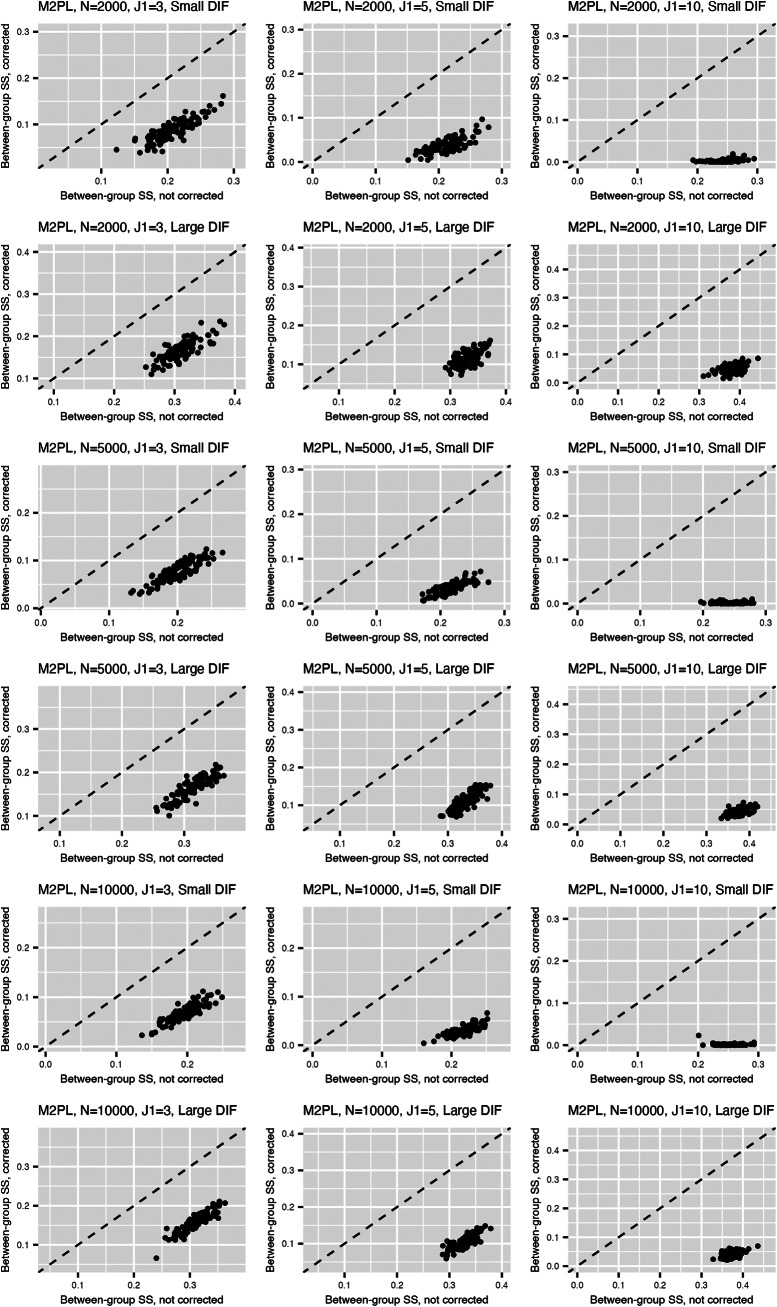
Between-group sum of squared bias for target trait estimation for the *M2PL* model with *uniform* DIF under different simulation settings. The *x*-axis corresponds to the estimation without DIF correction using the DIF items; the *y*-axis corresponds to the DIF-corrected estimation using the DIF items.

Figure 2 summarizes the values for the objective function (3) before and after adding the nuisance trait surrogate for the linear and M2PL models, with uniform DIF, 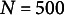 , and large DIF. For complete results of all simulation settings, see Figures B1 and B2 in the Appendix. We are able to minimize the objective function across simulation settings and replications. Specifically, we are able to obtain the zero of the objective function for the linear model. Tables 1 and 2 demonstrate the MSE of the item parameter estimates and the correlation between the ground truth and estimated nuisance traits. We observe that the MSE values are small in magnitude and the nuisance trait estimation correlation is high. It also shows that as the sample size increases, MSE tends to decrease and the nuisance trait correlation tends to increase. On the other hand, larger DIF effects and DIF item proportion lead to larger MSE. For the linear model, the nuisance trait correlation does not change with the sample size. For the M2PL model, it increases with the sample size. Figures B3 and B4 in the Appendix summarize the FI of the target trait 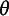 before and after adding the nuisance trait surrogate for the linear and M2PL models, respectively. We observe an increase in FI of the target trait in the measurement model after correcting for DIF in both models. Furthermore, Figure 3 compares the between-group SS bias of the corrected and not-corrected target trait estimates using the DIF items for the linear and M2PL models. The *x*-axis corresponds to the estimation without DIF correction, and the *y*-axis corresponds to the DIF-corrected estimation. The corresponding simulation setting is 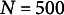 with large DIF; for the complete results, see Figures B5 and B6 in the Appendix. We see that the proposed method is able to correct for the target estimation bias introduced by DIF, as the between-group SS bias is significantly reduced after DIF correction for both models.

**Table 1:** Mean squared error of item parameter estimates and nuisance trait correlation for the *linear model* with *uniform* DIF under different simulation settings

		Small DIF			Large DIF		
		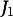 = 3	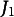 = 5	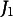 = 10	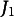 = 3	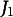 = 5	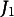 = 10
MSE (*d*)	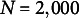	0.0365	0.0387	0.0363	0.1037	0.1055	0.0997
	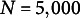	0.0377	0.0387	0.0375	0.1042	0.0969	0.1008
	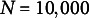	0.0375	0.0375	0.0377	0.1005	0.0999	0.0990
MSE ( 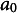 )	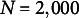	0.0014	0.0013	0.0014	0.0017	0.0020	0.0019
	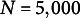	0.0007	0.0005	0.0006	0.0007	0.0007	0.0009
	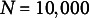	0.0003	0.0003	0.0004	0.0003	0.0004	0.0004
MSE ( 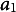 )	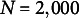	0.0494	0.0502	0.0495	0.1292	0.1344	0.1354
	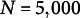	0.0497	0.0492	0.0493	0.1372	0.1335	0.1346
	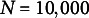	0.0489	0.0494	0.0498	0.1335	0.1350	0.1324
Corr ( 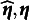 )	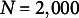	0.7112	0.7116	0.7125	0.7150	0.7103	0.7112
	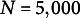	0.7115	0.7113	0.7140	0.7107	0.7107	0.7115
	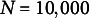	0.7162	0.7127	0.7128	0.7134	0.7110	0.7127

*Note*: The values are averaged across the DIF items and replications.

**Table 2:** Mean squared error of item parameter estimates and nuisance trait correlation for the *M2PL model* with *uniform* DIF under different simulation settings. The values are averaged across the DIF items and replications

		Small DIF			Large DIF		
		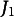 = 3	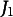 = 5	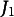 = 10	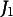 = 3	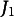 = 5	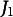 = 10
MSE (*d*)	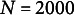	0.0466	0.0428	0.0307	0.0433	0.0411	0.0321
	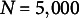	0.0440	0.0395	0.0269	0.0406	0.0384	0.0279
	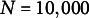	0.0378	0.0367	0.0252	0.0357	0.0360	0.0281
MSE ( 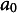 )	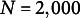	0.0108	0.0134	0.0403	0.0190	0.0244	0.0485
	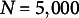	0.0102	0.0127	0.0482	0.0174	0.0258	0.0587
	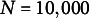	0.0083	0.0151	0.0472	0.0193	0.0232	0.0622
MSE ( 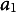 )	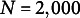	0.0366	0.0320	0.0387	0.0562	0.0545	0.0435
	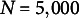	0.0128	0.0121	0.0140	0.0502	0.0460	0.0342
	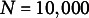	0.0087	0.0078	0.0085	0.0478	0.0419	0.0347
Corr ( 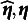 )	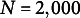	0.7515	0.7487	0.7635	0.8155	0.813	0.8175
	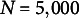	0.8196	0.8259	0.8246	0.8544	0.8513	0.8537
	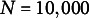	0.8447	0.8548	0.8557	0.8631	0.8707	0.8684

*Note*: The values are averaged across the DIF items and replications.

**Figure B7:**
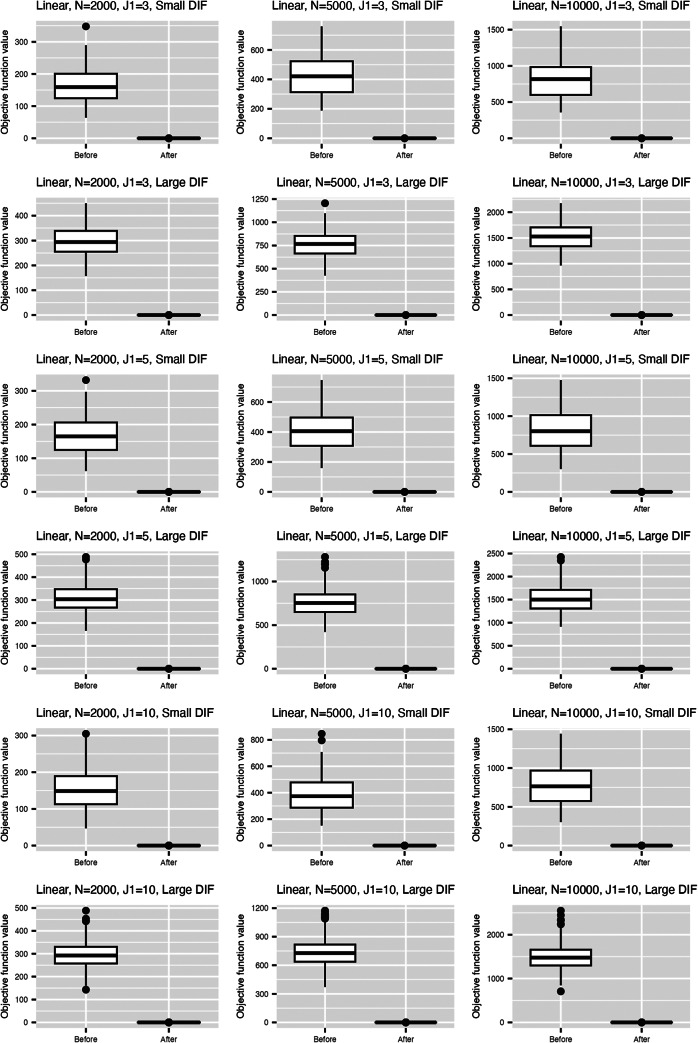
Objective function value before and after adding the nuisance trait surrogate for the *linear model* with *non-uniform* DIF under different simulation settings.

**Figure B8:**
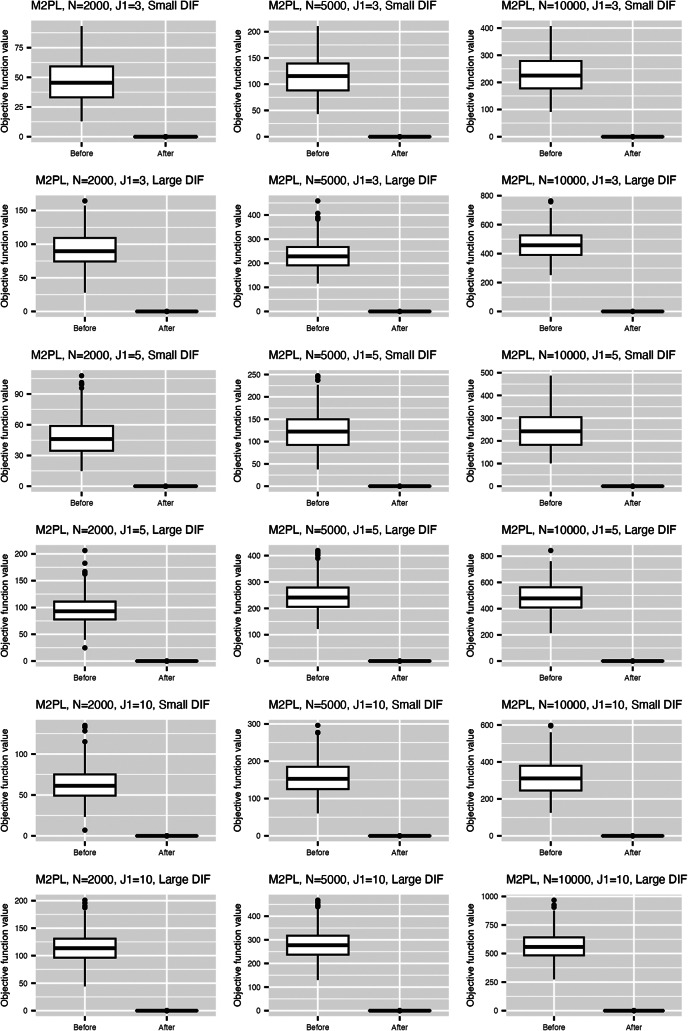
Objective function value before and after adding the nuisance trait surrogate for the *M2PL model* with *non-uniform* DIF under different simulation settings.

**Figure B9:**
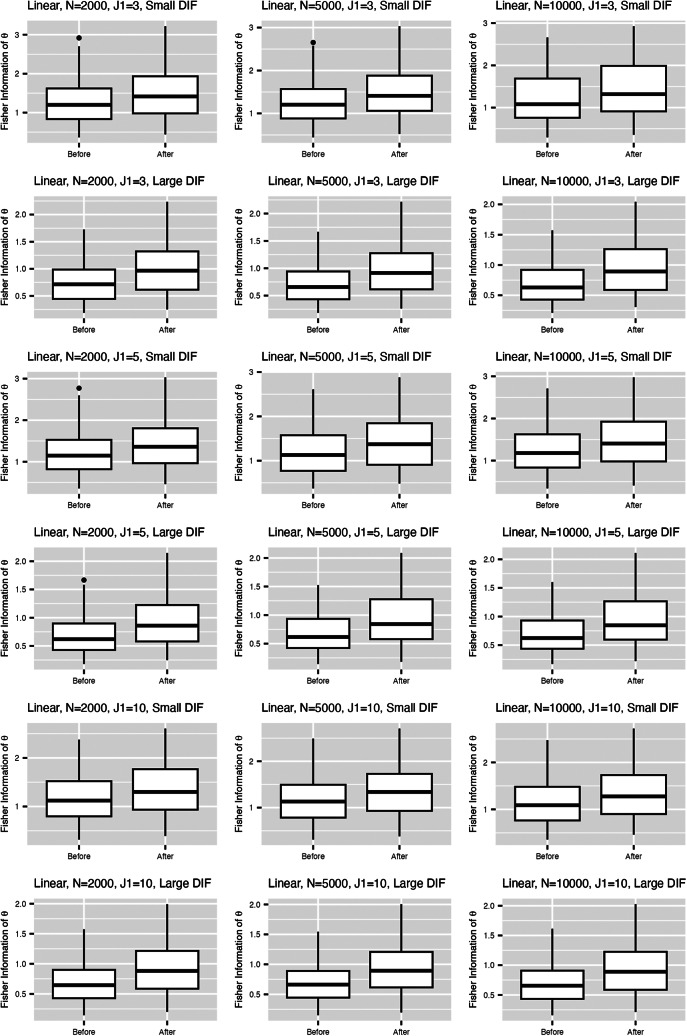
Fisher information of 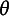 in the measurement model before and after adding the nuisance trait surrogate for the *linear* model with *non-uniform* DIF under different simulation settings.

**Figure B10:**
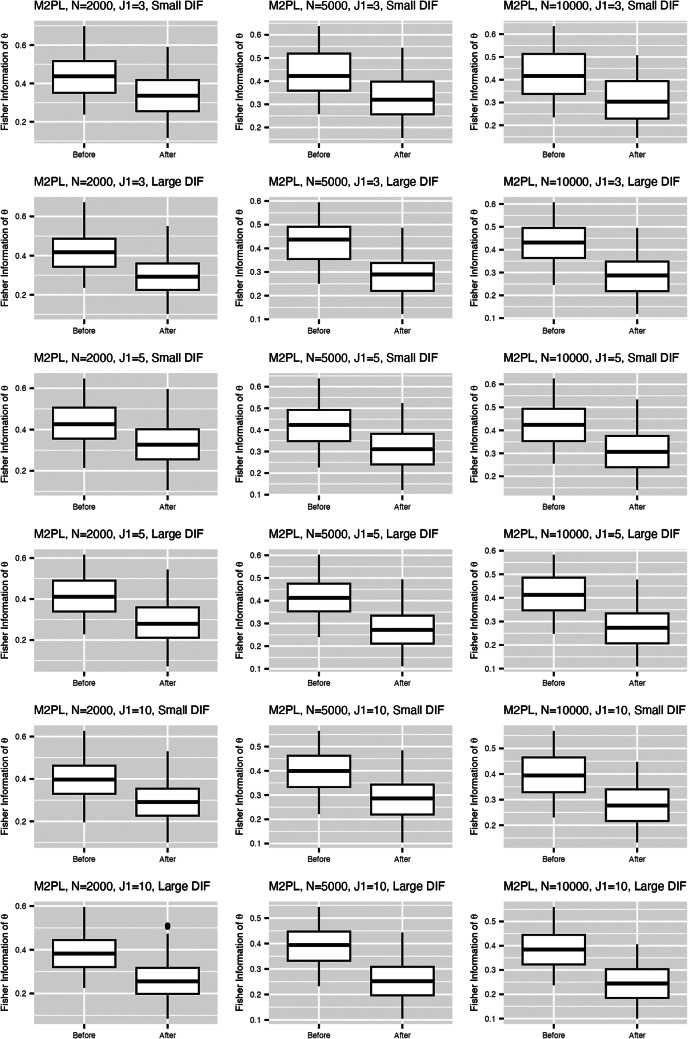
Fisher information of 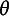 in the measurement model before and after adding the nuisance trait surrogate for the *M2PL* model with *non-uniform* DIF under different simulation settings.

**Figure B11:**
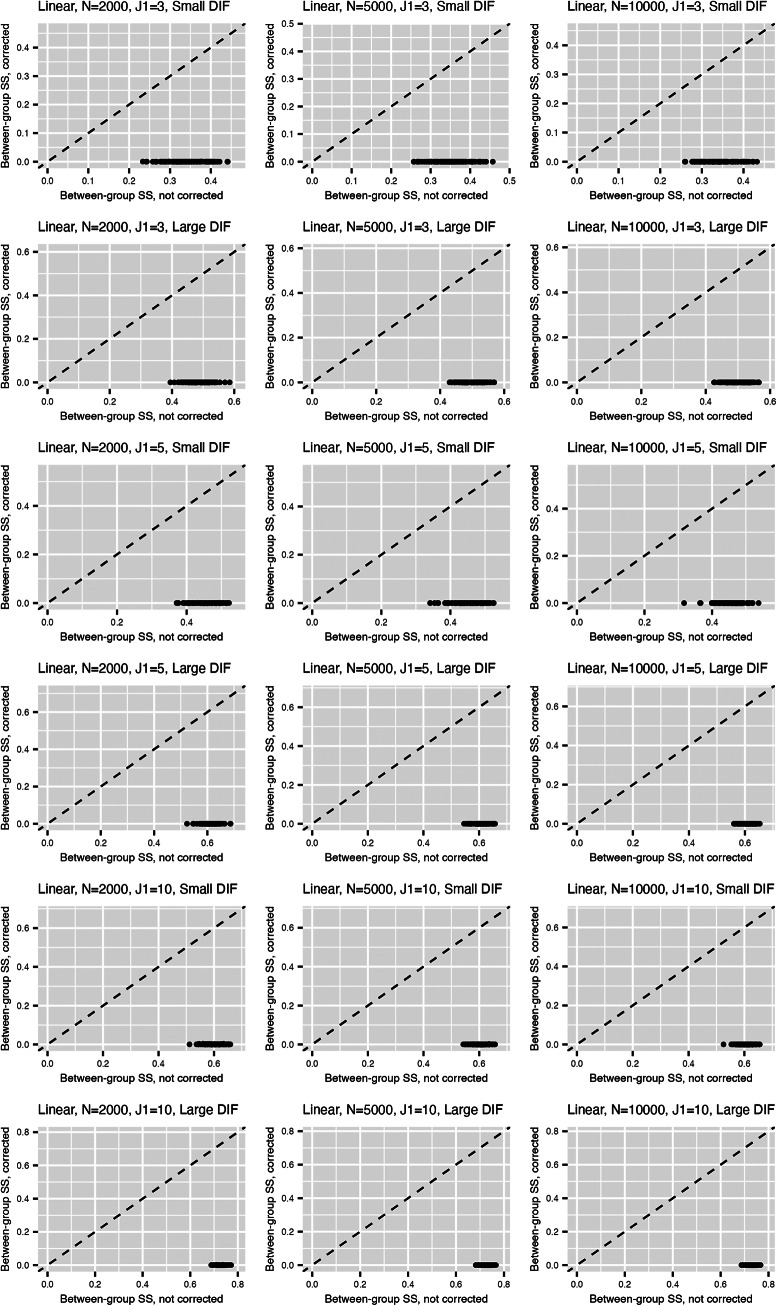
Between-group sum of squared bias for target trait estimation for the *linear* model with *non-uniform* DIF under different simulation settings. The *x*-axis corresponds to the estimation without DIF correction using the DIF items; the *y*-axis corresponds to the DIF-corrected estimation using the DIF items.

**Figure B12:**
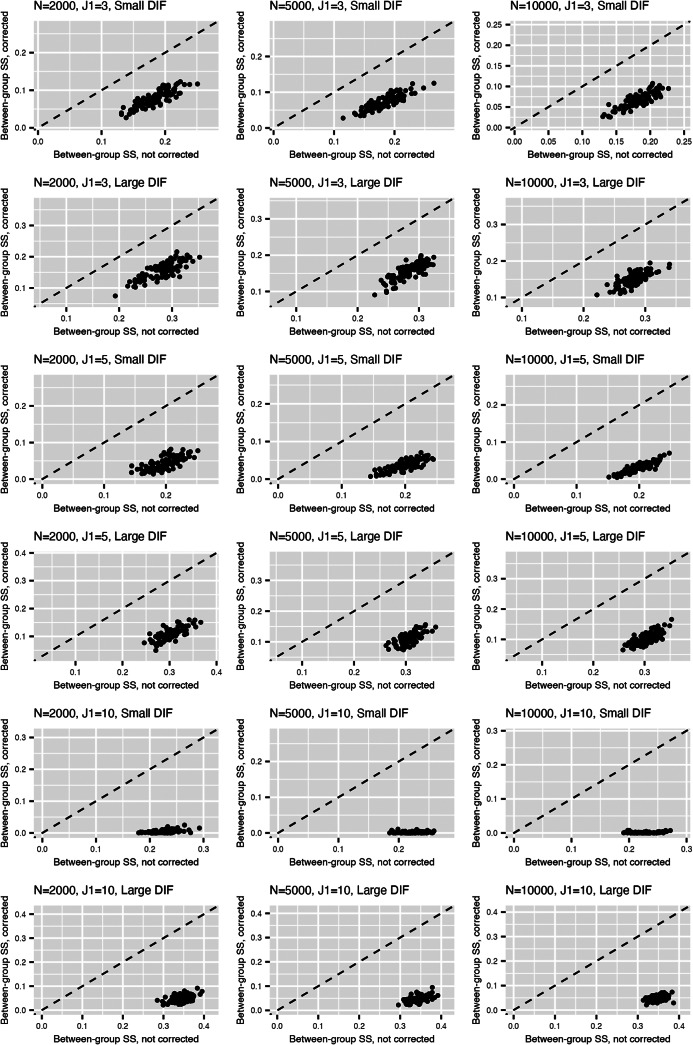
Between-group sum of squared bias for target trait estimation for the *M2PL* model with *non-uniform* DIF under different simulation settings. The *x*-axis corresponds to the estimation without DIF correction using the DIF items; the *y*-axis corresponds to the DIF-corrected estimation using the DIF items.

Results for non-uniform DIF are deferred to the Appendix. In Figures B7 and B8, we observe an increase in the minimized objective function values for non-uniform compared to uniform DIF, although the proposed method is still able to reduce DIF significantly. Similar results for item parameter and nuisance trait estimation, target trait FI, and between-group SS bias are observed (see Tables B1 and B2 and Figures B9–B12 in the Appendix).

## 4 Case study

We use the PIAAC 2012 survey data as a case study to demonstrate the effectiveness of our method. Response processes of 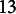 PSTRE items from 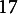 countries are considered in this study. PIAAC was the first attempt to assess the PSTRE construct on a large scale and as a single dimension. Under the PIAAC framework, PSTRE is defined as the use of digital technology, communication tools, and the internet to obtain and evaluate information, communicate with others, and perform practical tasks (OECD, 2012b). The survey also recorded a broad spectrum of respondents’ background information, such as gender, age, occupation, hourly income, education level, etc. To conduct DIF analysis, we consider age, income, and gender as the demographic grouping variables. We include the process data of 8,398 respondents who answered all 13 items and have no missing value of the three covariates in the study. For age, we use 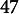 , which is the 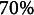 quantile, as the cutoff value to split the samples into younger and older sub-populations. The younger population is treated as the reference group, and the older population is treated as the focal group. For income, we first group the samples by their nationality, and then use the medium income of each nation as the cutoff value. The lower-income and higher-income groups are treated as the focal and the reference groups, respectively. For gender, female is treated as the focal group and male as the reference group.

Table 3 provides a descriptive summary of the 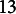 items, where *n* is the number of total possible actions, 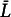 is the average process sequence length, and Correct 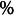 is the percentage receiving the full credit on each item. When solving for each item, the respondents are presented with one or more simulated informational and communicative (ICT) environments, such as an email inbox, a spreadsheet, a web browser, etc. For example, in item U01a, the respondents are presented with an email inbox interface and are asked to classify the email senders into “can come” and “cannot come” categories based on their email contents. To complete the task, the respondents need to conduct a sequence of clicking, dragging, or typing actions, which are recorded in the log files as process data. In addition, Tables B3 and B4 in the Appendix summarize the mean and standard deviation of the responses by group for each item, for the polytomous responses and binary responses, respectively.

**Table 3:** Summary statistics of 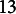 PIAAC problem-solving items

Item ID	Description	*n*	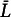	Correct %
U01a	Party invitations—Can/cannot come	51	17.2	59.8
U01b	Party invitations—Accommodations	55	26.1	52.4
U02	Meeting rooms	100	26.9	15.7
U03a	CD Tally	67	9.0	42.3
U04a	Class attendance	926	39.2	15.3
U06a	Sprained ankle—Site evaluation table	30	9.5	26.2
U06b	Sprained ankle—Reliable/trustworthy site	26	15.0	50.8
U07	Digital Photography Book Purchase	40	18.6	51.7
U11b	Locate E-mail—File 3 E-mails	137	24.8	26.5
U16	Reply all	886	32.4	63.9
U19a	Club membership—Member ID	162	17.3	75.1
U19b	Club membership—Eligibility for Club President	450	20.7	52.5
U23	Lamp return	164	21.7	38.2

*Note*: Here, *n* is the number of total possible actions, 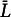 is the average process sequence length, and Correct 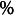 is the percentage of correct answers.

**Figure 4:**
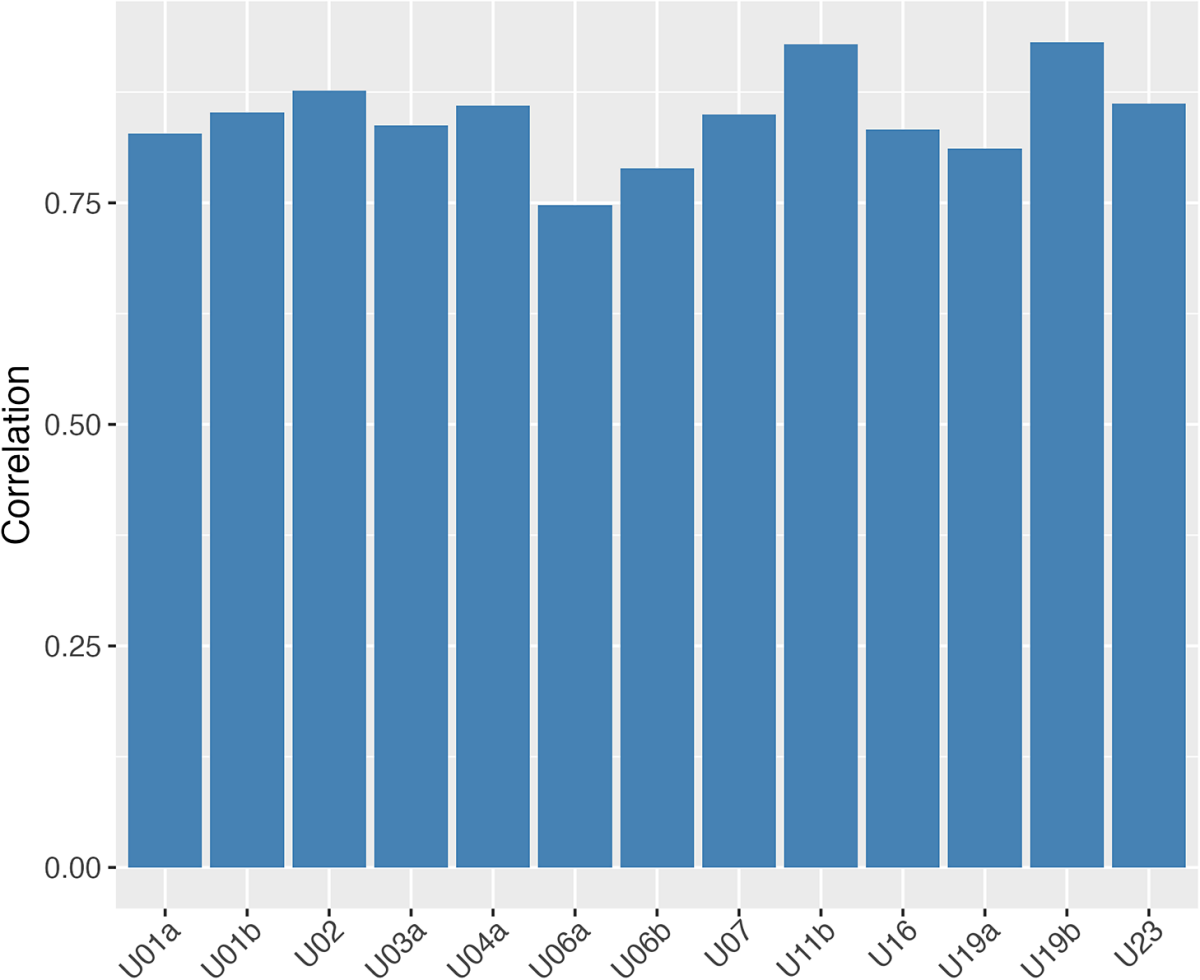
Out-of-sample prediction correlation of the process sequence length using the extracted features for each item.

We use the MDS approach proposed in Tang, Wang, He, et al. (2020) to extract item features that approximate the geometric distances defined by a dissimilarity matrix of the action sequences. We set the dimension of features to be 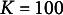 to ensure enough information is retained in the extracted features. To verify that the features contain an adequate amount of information in the process data, we use them to predict the responses with both linear regression and logistic regression. Results show that the extracted features can perfectly predict whether the examine received the full credit of each item with both regression methods. Engagement is often considered a nuisance trait in respondent behavior (Wise & DeMars, 2006; Wise & Kong, 2005), and one plausible measure of engagement is the length of a respondent’s process sequence. For each item, we randomly sample 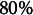 of training data to predict the process sequence length with the extracted features using ridge regression, where the ridge parameter is selected with cross-validation on the training data. Figure 4 demonstrates the out-of-sample correlation between the predicted and actual process sequence lengths for each item. It shows that the extracted features are able to predict the sequence length with high accuracy.

### 4.1 DIF existence

The proposed procedure assumes that we have access to the ground-truth target trait values. However, these target traits are unknown and need to be estimated in practice. To obtain initial estimates of the target traits, we utilize the responses to all 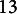 items. We then use these target trait estimates to identify DIF items. For the linear model, the latent traits are estimated by performing maximum-likelihood factor analysis on the response data. For the M2PL model, the link function 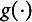 in Equation (1) is the logit function, and the reduced model without the nuisance trait is calibrated by maximizing the marginal likelihood using the expectation–maximization algorithm (Bock & Aitkin, 1981). The initial estimation 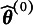 is the MLE estimate after item calibration.

Table 4 summarizes the DIF detection results using the initial trait estimate 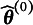 . For the age variable, 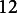 out of 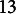 items are detected to have uniform DIF with both the linear model and the logistic model. For the income variable, five items have uniform DIF with both models. For the gender variable, 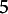 are uniform-DIF items with the linear model and 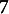 with the logistic model.

**Table 4:** DIF detection results *without* the nuisance trait

Item	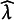 ( 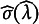 )					
	Linear model			Logistic model		
	Age	Income	Gender	Age	Income	Gender
U01a	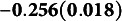	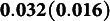	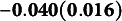	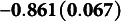	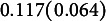	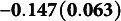
U01b	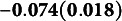	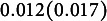	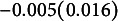	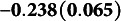	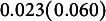	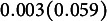
U02	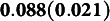	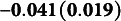	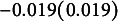	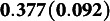	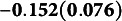	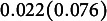
U03a	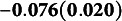	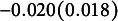	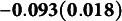	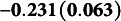	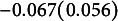	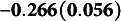
U04a	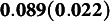	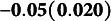	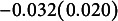	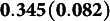	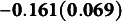	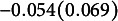
U06a	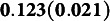	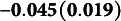	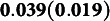	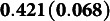	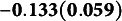	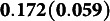
U06b	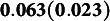	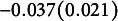	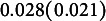	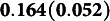	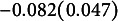	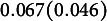
U07	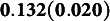	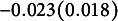	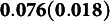	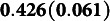	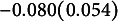	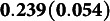
U11b	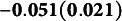	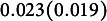	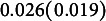	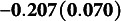	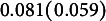	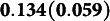
U16	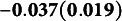	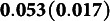	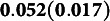	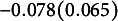	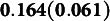	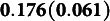
U19a	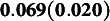	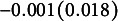	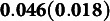	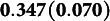	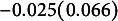	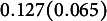
U19b	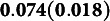	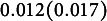	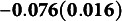	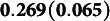	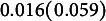	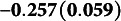
U23	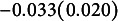	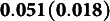	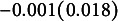	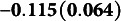	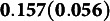	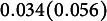

*Note*: Bold text indicates statistical significance under the 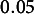 significance level.

### 4.2 DIF correction

We implement the proposed method to estimate the nuisance traits with the extracted process features, and to correct for DIF effects. For the linear model, we use the closed-form expression in Proposition 1 as the estimate, while for the M2PL model, the nuisance traits are estimated by solving (5) using the optim function with the L-BFGS-B optimizer in R. We also apply the iterative method in Section 2.5, the correlations between 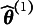 and 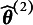 are over 99%.

**Figure 5:**
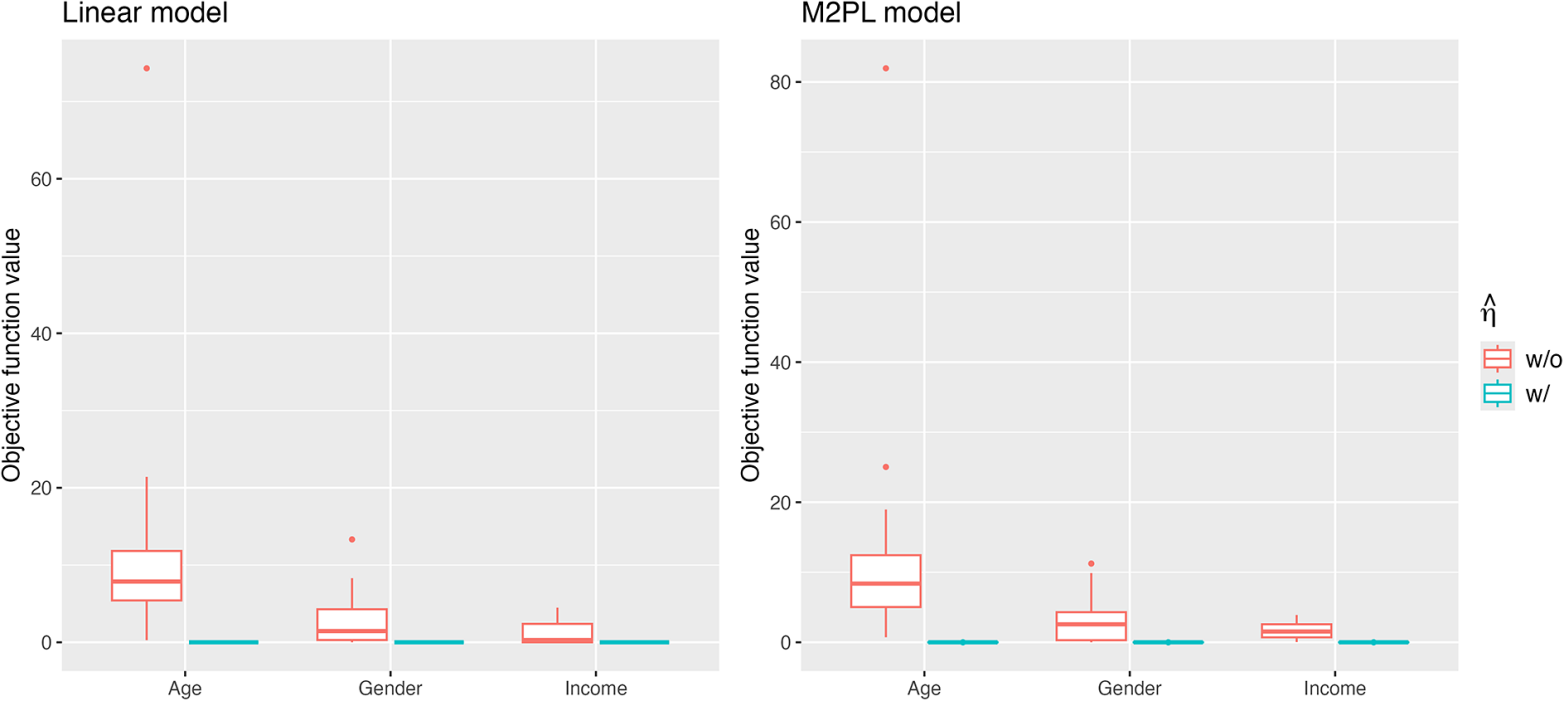
Comparing the objective function value with and without the nuisance trait surrogate for the linear model (left) and the M2PL model (right) with one grouping variable.

**Figure B13:**
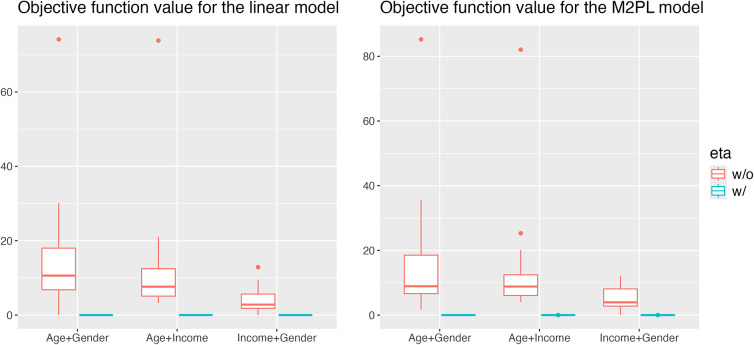
Comparing the objective function value with and without the nuisance trait surrogate for the linear (left) and the M2PL model (right) with two grouping variables.

Figure 5 includes the boxplots of the objective function (3) with and without the nuisance trait surrogate for the three grouping variables. We see that the estimated nuisance traits serve the purpose of minimizing the objective functions for both models. Table 5 demonstrates the sample mean FI of the target trait 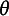 before and after adding the nuisance trait surrogate 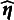 for items that exhibit DIF. We see that the FI increases in the linear model by adding the nuisance trait surrogate. For the M2PL model, we observe a moderate reduction for most items, and a significant reduction for items U06b and U07. For these two items, adding the nuisance trait surrogate significantly increases the prediction accuracy of the item response. The boxplots of the objective function with and without the nuisance trait when two grouping variables are present can be found in Figure B13 in the Appendix. We see that the estimated nuisance traits are able to minimize the objective function with two grouping variables. For the case study, we do not have access to the ground-truth target trait or a reliable unbiased estimator of the target trait as many items exhibit DIF. Therefore, we do not compare the between-group SS bias for the target trait estimates before and after DIF correction.

**Table 5:** Sample mean Fisher information for 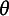 with and without nuisance trait surrogate, for three grouping variables and two models

	Linear model				M2PL model			
Item ID	w.o. 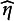	Age	Income	Gender	w.o. 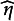	Age	Income	Gender
U01a	1.265	1.739	1.274	1.267	0.834	0.195	–	0.569
U01b	0.896	–	–	0.897	0.796	0.335	–	–
U02	1.183	1.231	1.294	–	0.756	0.729	0.462	–
U03a	0.623	–	0.627	0.684	0.667	0.168	–	0.176
U04a	0.312	0.337	0.322	–	0.395	0.193	0.183	–
U06a	0.345	0.397	0.361	0.351	0.489	0.146	0.285	0.248
U06b	0.133	0.137	–	–	0.146	0.030	–	0.022
U07	0.545	0.707	–	0.610	0.507	0.007	–	0.027
U11b	0.587	0.605	–	–	0.536	0.443	–	0.273
U16	0.822	0.827	0.851	0.840	0.645	–	0.512	0.292
U19a	0.573	0.587	–	0.584	0.507	0.340	–	0.426
U19b	1.520	–	1.523	–	0.750	0.143	0.625	–
U23	0.867	0.870	0.912	0.867	0.619	0.540	0.358	0.312

*Note*: Only the values corresponding to the DIF items are present.

**Figure 6:**
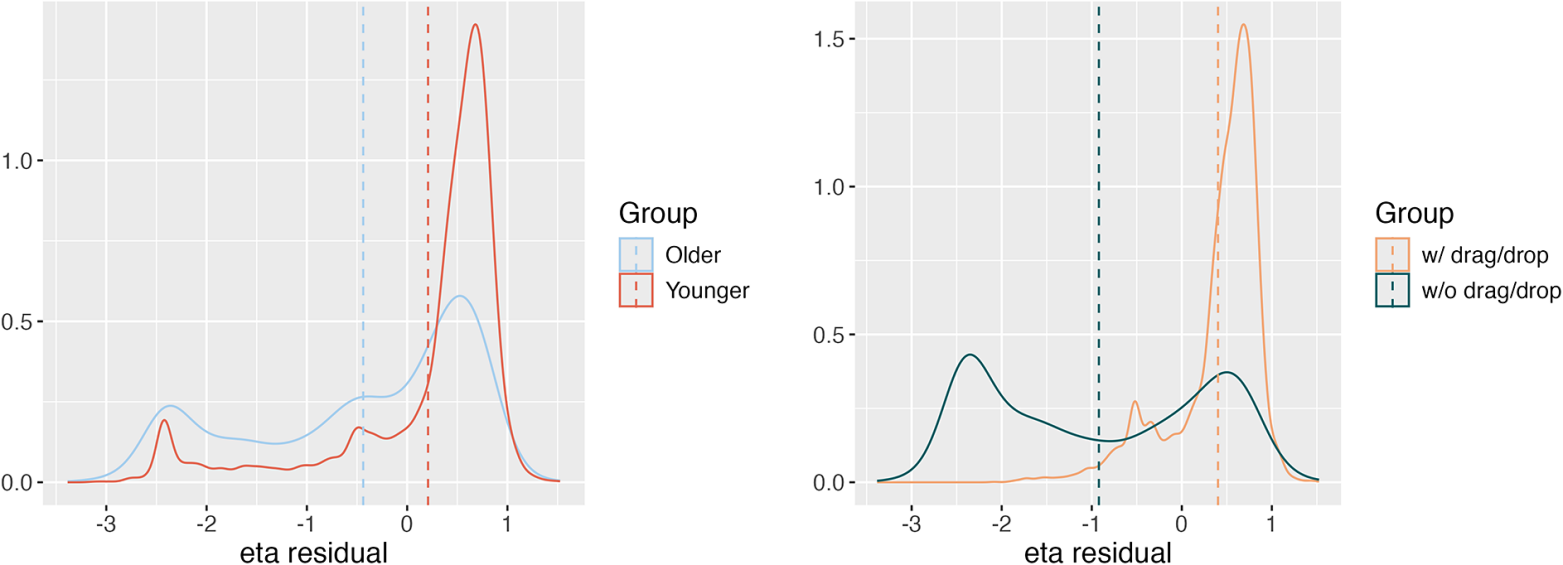
On the left: density plot of the residual nuisance trait 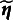 among the “old” group and the “young” group. On the right: density plot of the residual nuisance trait 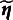 among the group that used drag/drop actions and those that did not.

As an illustration, we consider item U01a and the M2PL model to interpret the results for the age variable. After obtaining the estimated nuisance traits, we update the estimation of the target trait as 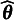 by solving Equation (6). Recall that DIF arises when the functioning of the response differs given the latent trait. Therefore, we study the characteristics of the residual nuisance trait given the target trait, i.e., 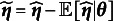 . To interpret why DIF occurs in item U01a, we check the original process sequences corresponding to the minimum and maximum values of 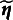 , and find that the usage of using drag/drop actions is related to the value of the residual nuisance trait. To verify this assumption, we calculate the correlation between 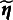 and whether drag/drop actions are used, which achieves 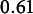 . And the correlation between 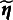 and the number of drag/drop actions achieves 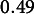 . These results suggest that the estimated nuisance variable can indicate the intensity of using drag/drop actions. Furthermore, the item response accuracy is 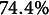 among the group that used drag/drop actions, versus 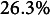 among those that did not use drag/drop actions. Figure 6 demonstrates the density plots of the residual nuisance trait 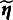 among the “older”/“younger” groups, and among the groups that did or did not use drag/drop actions. A possible interpretation of this phenomenon is that more senior individuals might be less familiar with this type of drag-and-drop mouse usage. It is also possibly more error-prone for more senior individuals to move emails using drag-and-drop actions because of the small font size in the email interface and the narrow distances between email folders. Among the individuals who used drag/drop actions, 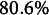 of the younger population had correct responses, while the percentage for the older population is only 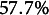 .

Notice that the majority of the items have some level of DIF. We apply the DIF correction method iteratively and found that the final estimates of 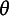 between iteration 1 and iteration 2 are over 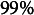 . To keep the illustration simple, we only report the results of iteration 1.

## 5 Discussion

Test fairness is a prominent concern within psychometric and educational research, and DIF analysis is a commonly practiced approach to ensure test fairness. When the distributions of the item response differ among two or more groups conditional on the target trait(s), DIF arises. Development of high-quality operational test items is costly, yet in practice, items identified with significant DIF effects are often discarded. In this article, we propose a method to “de-bias” items that are detected with DIF by incorporating data beyond item responses. We utilize the rich information contained in item process data, which capture the whole response processes of respondents when they interact with computer-based items. Specifically, we attribute DIF to multidimensionality, where nuisance traits with heterogeneous sub-group distributions also affect the item responses, besides the target trait to be measured. To uncover the unobserved nuisance trait, we propose to minimize the maximum-likelihood difference of the models with and without the grouping variable. In the simple case with linear regression models and one grouping variable, there is a closed-form solution to the proposed optimization problem. Simulation studies and a real data case study demonstrate the effectiveness of the proposed method.

Some limitations do exist in the current method. Firstly, the assumption that DIF stems from multidimensionality may not hold, as DIF may arise from other forms of model misspecification (Huang et al., 2024). Our study focuses on how process data help capture additional latent traits contributing to DIF within the multidimensionality framework. The amount of DIF reduction is limited to the portion that is caused by nuisance attributes and their predictability by the process data. Secondly, introducing the nuisance trait into the model might reduce measurement reliability, as suggested in some decrease in the FI for the target variable in the case study. It is of interest to study if a weighted summation of the maximum-likelihood reduction as in (3) and model liability quantification, such as the FI, would be appropriate as the new objective function. Thirdly, our proposed method relies on identifying a set of DIF-free or anchor items to identify the DIF items. In the case study, the initial targets are estimated assuming that all the items are DIF-free, and then utilized for DIF detection. However, this approach might be prone to bias when the influence of DIF on the initial trait estimation is significant. In the future, we are interested in more sophisticated DIF detection methods, such as item purification with stepwise model selection (Candell & Drasgow, 1988; Kopf et al., 2015a, 2015b). In addition, we can bypass the tedious iterative purification procedure by employing methods similar to the covariate-adjusted model with regularization (Ouyang et al., 2024; Wang, Zhu, et al., 2023), where the anchor item identification and latent trait estimation are carried out simultaneously. However, model identifiability must be carefully examined, as existing methods in the literature cannot be directly applied to our setting. Last but not least, process features are extracted from the action sequences and then utilized to linearly model the nuisance trait. However, a non-linear relationship between the nuisance trait and process data can be approximated by a neural network. Furthermore, it is generally difficult to systematically obtain interpretations of the nuisance traits. This is mostly because those traits are often unique to items and *Z* and thus not repeatedly measurable by multiple items. This is one of the limitations of the current method.

## Figures and Tables

**Figure A1:**
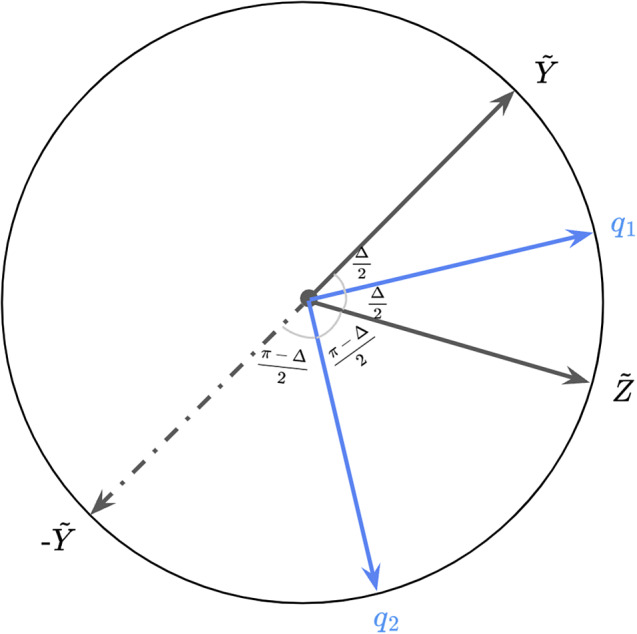
Geometric illustration for the proof of Proposition 1.

## Appendix A: Mathematical derivations

### A.1. Proof of Proposition 1.

Recall that  are, respectively, the residuals of  after regressing on  . Regress  on  :

and obtain the residuals  with  as the ordinary least squares (OLS) estimate. Specifically, since  with  and  , we have

(A.1)

Similarly, obtain the OLS estimates for (7):

(A.2)

and the residuals  for (8).

It is straightforward to show that

Therefore,

To obtain the zero of (9) is equivalent to obtaining the zero of  . Denote  and  . With  ,  , plugging in (A.1) and (A.2) yields

where  . In the above calculation, notice that  and  are numbers and therefore we could exchange order and take transpose when necessary. Consider the eigenvalue decomposition of  :  , where  ,  , and  . By the property of matrix  , there are closed-form expressions of its two eigen vectors and *K* eigenvalues. Specifically,

Under assumption (10), we have  . Let

and

then we have  and  .

We notice that  has more than one zero. We choose  over  because of the following geometric interpretations. We further write the scaled versions of  as  . Without loss of generality, we have assumed that  . We consider the case where  can almost perfectly predict  . Then,  , which leads to

(A.3)

Denote the angle between  as  , which is also approximately the angle between  . By assumption,  .

Rewrite  as

By (A.3), we also notice that

Therefore,

Therefore,  aligns with  and  aligns with  (see Figure A1 for geometric interpretations). Furthermore,  aligns with  , which is the projection of  onto the column space of  . Similarly,  aligns with the prediction of  by  . We choose to select  as the goal is to reduce DIF instead of using the process features to predict the response.

## Appendix B: Additional tables and figures

### B.2. Simulation with non-uniform DIF

**Table B1:** Mean squared error of item parameter estimates and nuisance trait correlation for the *linear model* with *non-uniform* DIF under different simulation settings

		Small DIF			Large DIF		
		=3	=5	=10	=3	=5	=10
MSE (*d*)		0.0325	0.0356	0.0328	0.0875	0.0886	0.0898
		0.0340	0.0319	0.0319	0.0882	0.0859	0.0882
		0.0328	0.0327	0.0319	0.0887	0.0859	0.0880
MSE ( )		0.0150	0.0142	0.0139	0.0380	0.0392	0.0395
		0.0147	0.0137	0.0146	0.0384	0.0398	0.0371
		0.0137	0.0135	0.0142	0.0384	0.0376	0.0362
MSE ( )		0.0682	0.0684	0.0658	0.1800	0.1772	0.1780
		0.0671	0.0641	0.0651	0.1783	0.1787	0.1777
		0.0639	0.0656	0.0648	0.1772	0.1762	0.1733
Corr ( )		0.6669	0.6667	0.6668	0.6628	0.6687	0.6684
		0.6684	0.6674	0.6679	0.6675	0.6676	0.6678
		0.6714	0.6694	0.6688	0.6687	0.6682	0.6710

*Note*: The values are averaged across the DIF items and replications.

**Table B2:** Mean squared error of item parameter estimates and nuisance trait correlation for the *M2PL model* with *non-uniform* DIF under different simulation settings

		Small DIF			Large DIF		
		=3	=5	=10	=3	=5	=10
MSE (*d*)		0.0451	0.0471	0.0331	0.0500	0.0487	0.0381
		0.0420	0.0386	0.0287	0.0438	0.0414	0.0335
		0.0403	0.0367	0.0273	0.0411	0.0401	0.0321
MSE ( )		0.0153	0.0142	0.0440	0.0200	0.0187	0.0469
		0.0097	0.0122	0.0465	0.0203	0.0204	0.0575
		0.0078	0.0136	0.0478	0.0202	0.0237	0.0568
MSE ( )		0.0360	0.0387	0.0436	0.0561	0.0605	0.0511
		0.0147	0.0134	0.0155	0.0508	0.0497	0.0400
		0.0112	0.0104	0.0093	0.0539	0.0539	0.0395
Corr ( )		0.7400	0.7408	0.7495	0.8075	0.8023	0.8083
		0.8115	0.8161	0.8115	0.8463	0.8467	0.8465
		0.8365	0.8417	0.8366	0.8602	0.8605	0.8605

*Note*: The values are averaged across the DIF items and replications.

### B.3. Case study

**Table B3:** The mean and standard deviation of the polytomous responses by group for each item

Item	Response mean (Response std)					
	Age		Income		Gender	
						
U01a	2.33 (1.13)	1.57 (1.34)	2.18 (1.2)	2.01 (1.29)	2.15 (1.24)	2.02 (1.26)
U01b	0.60 (0.49)	0.37 (0.48)	0.56 (0.50)	0.49 (0.50)	0.55 (0.50)	0.50 (0.50)
U02	1.03 (1.18)	0.63 (1.05)	1.03 (1.19)	0.80 (1.12)	0.97 (1.18)	0.84 (1.13)
U03a	0.50 (0.50)	0.29 (0.45)	0.47 (0.50)	0.40 (0.49)	0.47 (0.50)	0.39 (0.49)
U04a	0.67 (1.18)	0.47 (1.03)	0.70 (1.19)	0.53 (1.07)	0.66 (1.17)	0.56 (1.10)
U06a	0.29 (0.45)	0.21 (0.41)	0.30 (0.46)	0.23 (0.42)	0.27 (0.44)	0.26 (0.44)
U06b	0.53 (0.50)	0.46 (0.50)	0.54 (0.50)	0.48 (0.5)	0.51 (0.50)	0.51 (0.50)
U07	0.55 (0.50)	0.45 (0.50)	0.56 (0.50)	0.48 (0.50)	0.52 (0.50)	0.52 (0.50)
U11b	1.26 (1.36)	0.72 (1.18)	1.17 (1.34)	1.02 (1.31)	1.11 (1.34)	1.06 (1.32)
U16	0.70 (0.46)	0.50 (0.50)	0.66 (0.47)	0.62 (0.49)	0.65 (0.48)	0.63 (0.48)
U19a	0.79 (0.41)	0.67 (0.47)	0.78 (0.41)	0.73 (0.45)	0.76 (0.43)	0.74 (0.44)
U19b	1.33 (0.85)	1.00 (0.91)	1.30 (0.86)	1.16 (0.9)	1.29 (0.87)	1.16 (0.89)
U23	1.63 (1.38)	1.06 (1.33)	1.52 (1.39)	1.38 (1.39)	1.50 (1.39)	1.39 (1.39)

*Note*:  corresponds to the reference group, and  corresponds to the focal group.

**Table B4:** The mean and standard deviation of the binary responses by group for each item

Item	Response mean (Response std)					
	Age		Income		Gender	
						
U01a	0.70 (0.46)	0.39 (0.49)	0.63 (0.48)	0.57 (0.49)	0.63 (0.48)	0.57 (0.50)
U01b	0.60 (0.49)	0.37 (0.48)	0.56 (0.50)	0.49 (0.50)	0.55 (0.50)	0.50 (0.50)
U02	0.18 (0.38)	0.11 (0.31)	0.19 (0.39)	0.13 (0.34)	0.17 (0.38)	0.14 (0.35)
U03a	0.50 (0.50)	0.29 (0.45)	0.47 (0.50)	0.40 (0.49)	0.47 (0.50)	0.39 (0.49)
U04a	0.17 (0.38)	0.12 (0.32)	0.18 (0.38)	0.13 (0.34)	0.17 (0.37)	0.14 (0.35)
U06a	0.29 (0.45)	0.21 (0.41)	0.30 (0.46)	0.23 (0.42)	0.27 (0.44)	0.26 (0.44)
U06b	0.53 (0.50)	0.46 (0.50)	0.54 (0.50)	0.48 (0.50)	0.51 (0.50)	0.51 (0.50)
U07	0.55 (0.50)	0.45 (0.50)	0.56 (0.50)	0.48 (0.50)	0.52 (0.50)	0.52 (0.50)
U11b	0.31 (0.46)	0.16 (0.37)	0.29 (0.45)	0.25 (0.43)	0.27 (0.45)	0.26 (0.44)
U16	0.70 (0.46)	0.50 (0.50)	0.66 (0.47)	0.62 (0.49)	0.65 (0.48)	0.63 (0.48)
U19a	0.79 (0.41)	0.67 (0.47)	0.78 (0.41)	0.73 (0.45)	0.76 (0.43)	0.74 (0.44)
U19b	0.58 (0.49)	0.42 (0.49)	0.56 (0.5)	0.50 (0.50)	0.56 (0.50)	0.49 (0.50)
U23	0.44 (0.50)	0.26 (0.44)	0.40 (0.49)	0.37 (0.48)	0.40 (0.49)	0.36 (0.48)

*Note*:  corresponds to the reference group, and  corresponds to the focal group. To convert the polytomous responses to binary responses, we treat the full score as correct (  ), and other values as incorrect (  ).
